# Emotional Encoding Context Leads to Memory Bias in Individuals with High Anxiety

**DOI:** 10.3390/brainsci8010006

**Published:** 2017-12-27

**Authors:** Christopher Lee, Myra A. Fernandes

**Affiliations:** Department of Psychology, University of Waterloo, 200 University Ave. West, Waterloo, ON N2L 3G1, Canada; mafernan@uwaterloo.ca

**Keywords:** memory, anxiety, context, emotion, recognition

## Abstract

We investigated whether anxious individuals, who adopt an inherently negative mindset, demonstrate a particularly salient memory bias for words tainted by negative contexts. To this end, sequentially presented target words, overlayed onto negative or neutral pictures, were studied in separate blocks (within-subjects) using a deep or shallow encoding instruction (between-subjects). Following study, in Test 1, participants completed separate recognition test blocks for the words overlayed onto the negative and the neutral contexts. Following this, in Test 2, participants completed a recognition test for the foils from each Test 1 block. We found a significant three-way interaction on Test 2, such that individuals with high anxiety who initially studied target words using a shallow encoding instruction, demonstrated significantly elevated memory for foils that were contained within the negative Test 1 block. Results show that during retrieval (Test 1), participants re-entered the mode of processing (negative or neutral) engaged at encoding, tainting the encoding of foils with that same mode of processing. The findings suggest that individuals with high relative to low anxiety, adopt a particularly salient negative retrieval mode, and this creates a downstream bias in encoding and subsequent retrieval of otherwise neutral information.

## 1. Introduction

When trying to retrieve information about our past, it has been suggested that we do so by constraining our “memory search” to the mode of processing we had engaged during initial encoding [1]. Behavioural data from the memory-for-foils paradigm [1] supports the idea that a re-initiation of encoding-related processing occurs during a recognition test [2]. That is, if we focused on the meaning of a word during encoding, we think about word meanings again when we later search our memory to determine whether it was part of a previously studied list. Conversely, if we focused on the perceptual characteristics of the word (e.g., font colour) then subsequent memory searches will target perceptual aspects. A by-product of such a search strategy is that new information (such as foils, or distractors) that is present on that recognition test, inadvertently becomes encoded, and tainted with the same mode of processing, reaping the memory benefits (or costs) associated with that re-activated processing mode. In this study, we examined whether, during the process of remembering information learned within an emotionally negative relative to neutral context, task-irrelevant information gets encoded with that same emotional valence, resulting in its enhanced later memorability. The second aim of our study was to determine if the downstream memory bias, if any, would be more profound in those with high levels of generalised anxiety, as they have difficulty disengaging attention from negative information and mindsets [3,4,5]. 

### 1.1. Source-Constrained Retrieval and the Memory-for-Foils Effect

According to the transfer-appropriate processing principle [6], memory retrieval will be best when the processes invoked during retrieval match those undergone during encoding, consistent with these two processes being intimately linked and interdependent. In line with this, in the proceduralist perspective proposed by Kolers [7,8]—for review, see [9]—encoding and retrieval are seen as overlapping; as such, it becomes difficult, and potentially meaningless, to identify discrete encoding and retrieval phases.

One of the most compelling lines of research demonstrating a link between encoding and retrieval processes is that using the memory-for-foils paradigm developed by Jacoby and colleagues [1,10,11]. They proposed that when remembering previously encoded information, the mode of processing engaged during initial encoding is brought back online. In their research, they introduced a “memory-for-foils” paradigm in which participants initially studied separate lists of words either under a deep (pleasantness judgment) or shallow (vowel judgment) level of processing. Following study, two recognition tests were administered (Test 1), and participants were told that each corresponded to either the deeply or the shallowly studied target words, intermixed among unique sets of foil (lure) words. Finally, in a third phase, participants were given a surprise recognition test assessing memory for the foil words (Test 2). The critical finding from this paradigm was that participants displayed better recognition for foil words that were derived from the recognition test of deep target words than of shallow target words. 

To account for this effect, Jacoby and colleagues proposed that during retrieval (on Test 1) participants re-entered the original encoding mode (i.e., either deep encoding or shallow encoding) throughout that recognition test, so as to constrain their memory search. By re-engaging the encoding mode, at the time of retrieval, the same processing was applied to all words (both the old target words and the foils) on that recognition test. The end result was a benefit to memory on the subsequent recognition test (Test 2) for the foils initially contained within the “deep” recognition test, as compared to the “shallow” test. The benefit of deep processing had been conferred onto the foil words, thus producing a memory advantage for these foils.

If such a “source-constrained retrieval” is indeed occurring, then the “memory-for-foils” effect should be apparent following any number of different encoding manipulations (not just a level of processing one), as long as those modes of processing can be reinstated at the time of retrieval. There have been a number of corroborating studies since 2005, confirming and extending the memory-for-foils paradigm [12,13,14,15] and providing evidence consistent with the reinstatement of encoding processes at the time of retrieval. Importantly, Danckert, MacLeod, and Fernandes [2] showed that another encoding manipulation—other than the standard levels-of-processing manipulation—also produced the memory-for-foils pattern. In their study, imaging the referents of the words during encoding, as opposed to imaging the words transformed from lower to upper case, led to better memory for the foils (on Test 2) that came from the deep than shallow recognition test (i.e., from Test 1). 

### 1.2. Recapitulation and the Reactivation of Neural Representations of Emotional Information

In related work, initial cognitive processes or neural regions initially active at encoding have been shown to become re-engaged when tested at retrieval, in a process known as recapitulation [16,17]. In a recent study, Vogelsang, Bonnici, Bergstrom, Ranganath, and Simons [18] demonstrated the process of recapitulation using the memory-for-foils paradigm, during which they were in an fMRI scanner. Behavioural results replicated those of previous works. Interestingly, fMRI analysis also revealed that the left inferior frontal gyrus (a region active during a “deep encoding” instruction at encoding and not during a “shallow encoding” instruction) was active for foils that had been presented within a recognition test for deeply-encoded targets. Considering that this area was active during retrieval for deeply encoded words, but not for shallowly encoded words, suggests that constraining retrieval for a particular processing type does indeed reactivate that particular (deep) mode of processing. If the memory-for-foils paradigm can be used to induce recapitulation for words processed using a deep encoding instruction, this paradigm should also induce recapitulation for words encoded with an emotional mode of processing.

One aim of the current study was to determine whether negative visual contexts experienced during an encoding phase for targets could later produce enhanced memory for “foils” present during an initial test for that target information. Numerous studies have demonstrated a memory advantage for content that is of negative emotional valence, whether that content came in the form of pictures, statements, or words [19,20,21,22,23,24,25]. Given this, we reasoned that in the process of remembering information learned within a negative context, task-irrelevant information (the foils from the recognition test) would also be encoded and tagged with that same negative valence; the end result would be enhanced later memorability for these foils relative to foils encountered from a recognition test for neutral-context targets. 

In support of our reasoning, Bowen and Kensinger [26] have found evidence suggesting a neural explanation for the associated memory advantage. They investigated the influence of emotional valence on recapitulation by pairing neutral words with either negative, positive, or neutral faces or scenes. Participants then completed a recognition test (remember-know-new paradigm) for the words presented alone. Both encoding and retrieval were completed in an fMRI scanner. Results revealed greater overlap in neural regions for words that had been studied in a negative context, as compared to positive and neutral contexts, suggesting the negative mode of processing had been reactivated. The goal of our study was to determine if this reactivated emotional mode of processing would taint incidental information present at time of retrieval. 

### 1.3. Individual Differences in Anxiety and Memory Bias

The second aim of our study was to determine if the downstream memory bias, if any, would be more profound in those with high levels of anxiety. Among high trait anxiety groups, it has been suggested that negative, threat-relevant stimuli recruit significant attentional resources [27,28,29], and such groups are often characterized by intrusive or pervasive thoughts directed toward the source of anxiety [30]. Moreover, anxious individuals have demonstrated a tendency to attend to [31,32] and difficulty disengaging attention from [3,4,5] negative information. Research on mood-congruent memory [33] suggests that information is better remembered if one’s current mood matches the valence of the to-be-remembered information. As such, individuals plagued by an inherently negative mindset, such as highly anxious individuals, may have better memory for negative information. Consequently, this negative mindset and directed attention toward negative stimuli may influence the perception of incidental, otherwise neutral, stimuli in the current environment. In turn, negatively-tainted neutral information, such as foil words, may be easier for individuals with high, compared to low, anxiety to remember. In the current study, we adapted Jacoby’s memory for foils paradigm to (1) extend the source-constrained retrieval hypothesis to contrast effects derived from a negative versus neutral encoding context; and (2) to determine whether there are individual differences in the degree to which a negative or neutral context can influence subsequent memory for foils. To investigate the latter, we compared performance in individuals classified with high versus low levels of trait anxiety. Such a finding would extend those of other work [18,34] by suggesting that neutral information can become associated with, or tainted by, a negative context by virtue of invoking a constrained search of memory. As a consequence, the negatively-tainted information will be more memorable compared to information associated with a neutral context [19,20,21,23,24,25]. Following from previous work conducted by Jacoby and colleagues [1,10,11], we also instructed half of the participant group to study words using a deep encoding instruction and the other to study words using a shallow encoding instruction. As a result, our study consisted of two between groups variables, (1) trait anxiety groups; and (2) encoding instruction groups.

Based on past research, we anticipate a memory advantage on Test 1, for words studied in a negative context. Additionally, as a result of being associated with the negative information, a memory advantage may be conferred on the foil words within the negative Test 1 subtest block. As a consequence, recognition performance for the negative foil words, assessed on Test 2, will be higher than for foils derived from the neutral Test 1 subtest. 

## 2. Materials and Methods

### 2.1. Participants

Eighty undergraduate students from the University of Waterloo (64 females) completed the experiment for partial course credit. Forty participants were randomly assigned to a deep encoding instruction group (mean age = 19.98 years, SD = 2.03, range = 16–26) and 40 were randomly assigned to a shallow encoding group (mean age = 20.18 years, SD = 4.50, range = 17–46). Participants were recruited in accordance with ethical guidelines governing the use of humans in research.

All participants completed the Depression Anxiety Stress Scales (DASS) [35]. The scales contains 42 items, 14 of which are dedicated to measuring each separate construct. An individual item consists of a statement that participants are instructed to provide a rating indicating how much the statement relates to them over the past week. Ratings are made on a 4-point scale ranging from 0 to 3, where 0 represents no relation to the statement, and 3 indicates that the statement relates to them a lot. 

Participants were also divided into two separate groups based on a median of the anxiety ratings for the sample. The median score for anxiety was 7.5 (SD = 8.27). Median scores for depression and stress were 4 (SD = 9.18) and 11.5 (SD = 8.99), respectively. Our “high anxiety” group consisted of participants with mean anxiety scores higher than 7.5 (deep encoding group *N* = 20, mean anxiety = 15.90, SD = 7.5; shallow encoding group *N* = 20, mean anxiety = 14.65, SD = 8.05), and the remainder were categorized into the “low anxiety” group (deep encoding group *N* = 20, mean anxiety = 3.20, SD = 2.09; shallow encoding group *N* = 20, mean anxiety = 3.35, SD = 2.49). Note that independent sample t-tests revealed no significant difference between the high anxiety (*t*(38) = 0.51, *p* = 0.62) groups who studied words under either a deep or shallow encoding instruction, nor was there a difference between the low anxiety (*t*(38) = 0.21, *p* = 0.84) groups.

We conducted independent sample t-tests to determine whether the high and low anxiety groups differed on depression or stress as reported on the DASS. There was a significant difference in mean depression (*t*(78) = 5.31, *p* < 0.001), whereby the high anxiety group (M = 12.83, SD = 10.26) reported a higher depression level than the low anxiety group (M = 3.43, SD = 4.44). Similarly, there was a significant difference in trait stress levels (*t*(78) = 5.51, *p* < 0.001), whereby the high anxiety group (M = 17.73, SD = 8.84) reported a higher level of stress than the low anxiety group (M = 8.28, SD = 6.30).

### 2.2. Materials

#### 2.2.1. Photos Used as Visual Context

**Figure 1:**
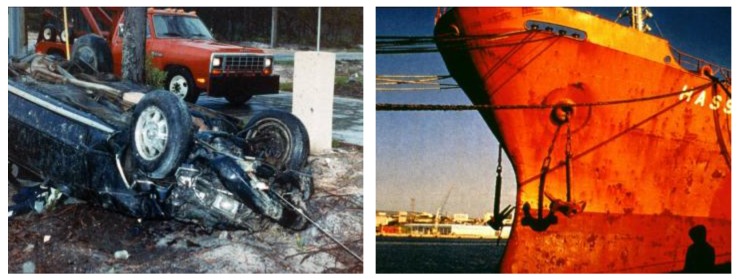
Sample IAPS picture stimuli of negative (**left**) and neutral (**right**) context images.

Pictures used as background visual context were taken from the International Affective Picture System (IAPS) [36]. The IAPS is a database of pictures with normative ratings for valence, arousal, and dominance based on a 9-point Likert scale. Two sets of pictures were created for the study. One set consisted of 36 pictures that were negative in valence (M = 2.51, SD = 0.46) and the other set consisted of 36 pictures that were neutral in valence (M = 5.06, SD = 0.56). Normative arousal ratings were matched across the negative (M = 5.01, SD = 0.63) and neutral pictures sets (M = 4.86, SD = 0.73). The selected pictures were re-sized to the dimensions of 11.7 cm × 8.8 cm from their original size of 26 cm × 19.5 cm to fit on the computer monitor. See Figure 1 for sample context picture stimuli.

#### 2.2.2. Words Used as Targets and Foils

Neutral words were selected from the Affective Norms for English Words database [37]. The ANEW consists of a set of English words with normative ratings for valence, arousal, and dominance. All words selected from the database were concrete nouns, and neutral in valence. Two unique 36-item word lists were created as the study lists. For Test 1, each of these lists was intermixed among a different set of 36 foil words in separate subtests. A separate, unique set of 72 foils was used as lure words on Test 2. Word lists were matched on normative valence and arousal ratings across the negative (valence M = 5.7, SD = 1.24; arousal M = 4.71, SD = 0.87), and neutral (valence M = 5.42, SD = 1.37; arousal M = 4.79, SD = 0.82) target lists, and the negative (valence M = 5.77, SD = 0.96, arousal M = 4.52, SD = 0.87) and neutral (valence M = 5.6, SD = 0.86; arousal M = 4.36, SD = 0.86) foil lists. 

### 2.3. Procedure

Participants completed the experiment individually using a desktop computer (19 inch monitor). Stimulus presentation and response recording were controlled using E-Prime 2.0 (Psychology Software Tools, Inc., Pittsburgh, PA, USA) [38]. Participants provided signed consent before providing demographic information, including age, gender, years of education, and age that the participant had learned English. Participants then completed the DASS (Psychology Foundation of Australia, Sydney, Australia) [35] either as the first or final phase of the experiment to ensure the scales had no influence on test responses. For this scale, each item was displayed on the computer monitor. Participants were given a total of 5 s to respond to each statement with a keypress indicating their endorsement of the statement. 

The study consisted of a 2 × 2 × 2 design, with Encoding Context Valence (negative or neutral) as a within-participant factor, and Encoding Instruction Group (shallow or deep) and Anxiety Group (low or high) as between-participant factors.

#### 2.3.1. Encoding Phase

Participants were presented with a set of 72 words (in 105 point white Sans font with a 0.14 cm black border) overlayed onto individual IAPS pictures (26 cm by 19.5 cm) in sequential trials, and were informed that their memory for the words would later be assessed. The 72 word-picture set was divided into 2 separate sublists, one for words overlayed onto negative IAPS pictures and another for words overlayed onto neutral IAPS pictures. Henceforth, these sublists will be referred to as “negative study words” and “neutral study words”. The order in which the sublists were studied was counterbalanced across participants. For each trial, participants were instructed to study each word and to respond to two questions that appeared below the word-context pair, one at a time. For those in the shallow encoding group the first question asked them to indicate whether the word displayed on that trial contained the vowel “a”. For the deep encoding group, the first question asked them to indicate whether the word displayed onscreen represented an object that was living or non-living. Responses for each group were made by pressing the A or D key on a QWERTY keyboard. To avoid the possibility that participants could evade or not attend to the picture that was used as a context, participants in both encoding groups also answered a matching question; they indicated, subjectively, whether the word represented an object similar to the contents of the picture (by pressing the 4 or 6 key on the number pad of a QWERTY keyboard). Each question remained on the screen for 4 s, regardless of when the participant responded, thus providing 8 s in total, per study trial. A fixation cross was displayed between each trial for 250 ms. 

#### 2.3.2. Intentional Recognition Test 1

Following the Encoding Phase, participants completed an intentional recognition test for words presented alone (i.e., without picture context), divided into two subtests. Each subtest contained either the negative study words or neutral study words as targets, intermixed among a unique set of foil words. Thus, each subtest contained a total of 72 words (36 studied targets and 36 foils). The subtest containing the negative study words will be referred to as “negative recognition test”; and the subtest containing the neutral study words will be referred to as “neutral recognition test”. On each recognition test trial, a word (in white Courier New font at 26 point) was shown in the centre of the computer monitor. Participants were instructed to determine whether the word was “old” or “new”, by pressing the J or L key on a QWERTY keyboard. Each word remained on the screen until a response was provided, followed immediately by the subsequent target word. Prior to beginning a subtest, participants were told whether the target words were derived from either the sublist of negative study words or of neutral study words. The order in which each recognition subtest was completed was counterbalanced across participants.

#### 2.3.3. Incidental Recognition Test 2

A surprise recognition test was subsequently administered for the 36 foil words (same font and size as at encoding) from the negative and the 36 from the neutral recognition tests. These 72 foil words were intermixed among a brand new set of 72 foils, thus forming a recognition test totalling 144 words. Participants were told to identify a word as “old” if they remembered seeing it in the previous phase, or “new” (never seen in this experiment), by pressing the J or L key on a QWERTY keyboard. 

## 3. Results

For Test 1, accuracy rates for target word memory were derived from the negative and neutral study sublists. Accuracy was calculated as hit rate (total hits divided by 36) minus false alarm rate (total false alarms divided by 36). For Test 2, accuracy rates were calculated as hit rate for foil words derived from the negative or neutral Test 1 subtest (total hits for each separate recognition test divided by 36) minus the overall false alarm rate on Test 2 (total false alarms divided by 72). 

### 3.1. Test 1: Intentional Recognition of Studied Targets

We conducted a 2 (Recognition Test Valence: negative, neutral contexts) × 2 (Anxiety Groups: high, low trait anxiety groups) × 2 (Encoding Instruction Group: deep, shallow encoding groups) repeated measures ANOVA on accuracy rates for Test 1. Main effects were non-significant for Recognition Test Valence, Anxiety Group, or Encoding Instruction Group. Two-way and 3-way interactions were also non-significant. See Table 1 for mean hit, false alarm and accuracy rates.

### 3.2. Test 2: Incidental Memory for Foils

We conducted a 2 (Foil Valence: foils derived from the negative or neutral Test 1 blocks) × 2 (Anxiety Groups: high, low trait anxiety groups) × 2 (Encoding Instruction Group: deep, shallow encoding groups) repeated measures ANOVA on accuracy rate for Test 2 (see Table 2 for means). All main effects were non-significant. All 2-way interactions were also non-significant. There was, however, a significant 3-way interaction (*F*(1, 76) = 4.46, MSE < 0.01, ηp^2^ = 0.06, *p* < 0.04).

**Figure 2:**
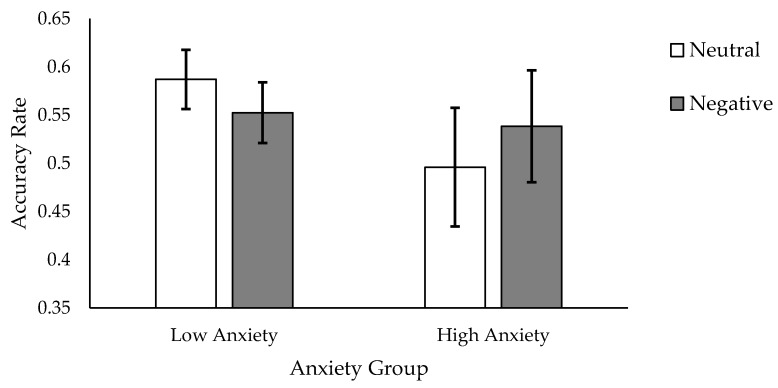
Mean accuracy rates on Test 2 for foils derived from the neutral and negative Test 1 subtests for low and high anxiety groups, following shallow encoding of targets. Error bars show the standard error.

For the high anxiety group, when words were studied using a shallow encoding instruction, participants demonstrated a higher recognition accuracy for foils derived from the negative (M = 0.54, SD = 0.26) recognition subtest, compared to the neutral (M = 0.50, SD = 0.28) subtest. The low anxiety group showed the opposite pattern, with accuracy being higher for foils derived from the neutral (M = 0.59, SD = 0.14) than negative (M = 0.55, SD = 0.14) subtest (see Figure 2).

When words were studied using a deep mode of processing, however, the pattern of memory for negative versus neutral foils did not differ reliably across anxiety groups.

## 4. Discussion

In this study, we examined whether encoding of target words presented within a negative visual context could create a downstream recognition memory bias. We also assessed whether there were individual differences in the pattern of memory bias, particularly in those with high levels of anxiety, who are believed to be particularly sensitive to negative mindsets [30,39]. We found such a bias, indexed by memory for foils on Test 2. That is, we found a significant Foil Valence × Anxiety Group × Encoding Instruction interaction: high trait anxiety participants who had studied words using a shallow encoding instruction demonstrated a higher accuracy rate for the “negatively-tinged” than the “neutrally-tinged” foils. 

Results support both of our hypotheses. In cases where words were encoded shallowly, participants displayed higher recognition for target foil words initially encountered amongst target words learned in a negative context, though this memory advantage was seen only in participants with high trait anxiety. Findings from the study suggest that initial encoding context can indeed create a downstream memory bias, extending the source-constrained retrieval hypothesis to emotional contexts. Our results also suggest that negative encoding contexts produce a particularly salient bias for individuals characterised by high levels of anxiety. That is, memory for otherwise neutral stimuli, particularly among high anxiety individuals, may become grossly tainted by the mode of processing engaged when such stimuli are incidentally encountered. In being tainted by a negative mode of processing, or incidental stimuli (the foil words in our study), became more memorable.

Support for our interpretation comes from other studies showing that neutral stimuli incidentally presented, or associated with, a negative or threatening context can become tainted [40]. Otherwise neutral stimuli can indeed become affiliated with the retrieved threat-related information. As suggested by past related work, any memory enhancement that the individual harboured for the original threat then transfers to the neutral stimuli, making these incidental stimuli more memorable as well [26,34]. Notably, in our study, the downstream memory bias for “negatively-tinged” foils was only demonstrated by participants with high trait anxiety. One possible explanation for this finding is that the amount of attentional resources directed toward threat-relevant or threat-related stimuli differ in those with high versus low anxiety. For high anxiety individuals, it has been shown that threat-relevant stimuli recruit significant attentional [27,28,29] and working memory [41] resources. In being tainted by the negative context, the otherwise neutral stimuli may now occupy more working memory space, according for their enhanced memorability. In contrast to the high anxiety group, the low anxiety individuals showed the exact opposite pattern of effects on Test 2, whereby neutrally-tainted foil words were more salient than negatively-tainted foils. This pattern may have occurred because Test 2 consisted of intermixed trial types (both negative and neutral); another way to interpret our results is that the salience of the neutral foils relative to the negative ones was enhanced for those with low compared to high anxiety. That is, when memory for the set of foils was examined, the low anxiety individuals preferentially accessed the neutral ones. In contrast, high anxiety individuals preferentially searched for, or distributed their attention towards, sources of threat, accounting for their higher memory for the negative over neutral foils.

Previous research has explored constrained memory search for words that were encoded with various levels of processing [1,2,10]. In these studies, participants demonstrated a recognition bias for foils derived from tests of words that had initially been processed deeply, as opposed to shallowly. In our study, however, we manipulated the level of processing across groups, and failed to find a main effect of depth of processing on Test 2 performance. In fact, there were no differences between the groups until Anxiety Group and Foil Valence were taken into consideration. Unlike many other studies exploring constrained memory search [2,12,13,14,15], our study implemented an emotional context manipulation in conjunction with a depth of processing instruction at the time of encoding. The discrepancy between the findings in our study and those of prior works suggests that the inclusion of an emotional context at encoding altered the eventual downstream memory bias, reducing any effect from depth of processing alone. One possible explanation is that there is a maximal amount of benefit a target can receive from a particular encoding strategy. As a consequence of studying words using a deep encoding instruction, the further benefit offered by negative emotional valence could not be observed (i.e., memory performance was already high). In contrast to the deep instruction, a shallow mode of processing offers relatively less benefit to memory. Here the negative contexts had more leeway to provide an observable benefit. 

Another possible explanation is that processing words using a deep rather than shallow encoding instruction diverts attention away from the emotional context. A deep encoding instruction asks the participant to purposely think of the meaning of that target word, and focuses attention and cognitive thought on other items/words associated with the word’s meaning. Conversely, words encoded using a shallow instruction are less likely to link to semantic associations as the focus of attention is instead on decoding the physical features of the lettering used to spell it (i.e., whether there is the vowel “a” in the word). This may allow more availability of resources to be devoted to processing the valence of the underlying visual image. Importantly it was only those with high, and not low, anxiety who were sensitive to the biasing effect of the negatively valenced image.

One aspect of our data which requires further explanation, however, is the absence of the expected memory advantage for negative target words on Test 1. The lack of effect may have occurred because our chosen visual negative contexts produced inconsistent affective responses across participants. That is, in our study, the images used as negative contexts consisted of a wide variety of content (e.g., blood, cemeteries, automobile accidents). As suggested by Radomsky and Rachman [42], a memory advantage for emotional material might only be evident with stimuli that are particularly threatening to that individual. Due to the variety of negative images used in our study, a given participant might have considered one picture threatening, stimulating a high affective response, whereas another participant might not have perceived the same image as a threat. For example, one participant may be upset by the sight of blood shown in some of the pictures, whereas others may not. We should not expect a memory advantage for a word accompanied by a non-threatening picture, since the taint of a non-threatening picture would effectively be similar to that from a neutral picture. Future studies could examine a possible downstream memory effect when the visual context at study is more consistently related to a specific threat, in subgroups of anxious participants (e.g., spider pictures in spider-phobics). 

Why then was there a downstream effect on memory for the foils? Guez and Naveh-Benjamin [43] suggest that the influence of a particular manipulation on memory (in their case divided attention, and here, emotional context) may become more pronounced over longer time frames, as consolidation of the memory takes place. Given the relatively short amount of elapsed time between study and Test 1 in our paradigm, the benefit conferred to targets overlayed onto negative versus neutral contexts may not have yet emerged. That is, memory for the neutral-context targets was high, though if we had delayed Test 1, it is possible that differential forgetting may have occurred, allowing us to see the expected memorial benefit for targets encoded within a negative context. Given our paradigm, it is therefore even more remarkable that we observed the memory for foils effect on Test 2, for the foils initially encountered during the negative Test 1 subtest. Clearly our manipulation of context produced some difference in how the “negative-context” versus “neutral-context” targets were subsequently evaluated, and this differential evaluation was inadvertently applied to the foils contained on each test, resulting in the differential memory for foils. Alternatively, participants may not have encoded as deeply or with as salient of an emotional tag during our encoding phase due to the pictures being presented in the background. However, the categorical membership of all the words in Test 1 as negative or neutral was likely reinforced through our instructions for Test 1, in which we informed the participant they would perform a recognition test for either the words overlayed onto negative or neutral pictures. When completing Test 1, informing participants of the origin of the set of words in each subtest may have brought the images back to mind, contributing to the subsequent emotionality boost we observed on Test 2 for the foils.

## 5. Conclusions

Results support our hypothesis that initial encoding context (whether negative or neutral) can influence later memory for incidental stimuli (the foils). In the framework of our research question, emotional events from the past can taint our perception of the present, making current circumstances more memorable. When we constrain our memory search to information or events encountered within a negative context, or learnt using a negative mode of processing, some memory benefit held by those thoughts may be conferred unto incidental stimuli within our current environment. Of note, this downstream memory bias was significant only in individuals with high levels of trait anxiety. Our findings suggest that anxiety can engender a mode of cognitive processing that taints or colours otherwise neutral information. 

## Figures and Tables

**Table 1:** Test 1: Memory Performance for Participants with High versus Low Trait Anxiety in the Deep and Shallow Encoding Conditions.

	Low Anxiety Group		High Anxiety Group	
	Deep	Shallow	Deep	Shallow
Hit Rate				
Negative	0.69 (0.21)	0.71 (0.15)	0.70 (0.18)	0.70 (0.13)
Neutral	0.73 (0.20)	0.68 (0.11)	0.68 (0.24)	0.70 (0.18)
False Alarm Rate				
Negative	0.14 (0.18)	0.08 (0.07)	0.15 (0.24)	0.14 (0.21)
Neutral	0.10 (0.19)	0.06 (0.05)	0.19 (0.33)	0.12 (0.21)
Accuracy Rate				
Negative	0.55 (0.34)	0.64 (0.16)	0.55 (0.40)	0.56 (0.28)
Neutral	0.63 (0.35)	0.62 (0.11)	0.50 (0.55)	0.59 (0.28)

**Table 2:** Test 2: Memory for Foils and False Alarm Rate for Participants with High versus Low Trait Anxiety in the Deep and Shallow Encoding Condition.

	Low Anxiety Group		High Anxiety Group	
	Deep	Shallow	Deep	Shallow
Hit Rate				
Negative	0.78 (0.17)	0.78 (0.18)	0.72 (0.19)	0.75 (0.25)
Neutral	0.76 (0.16)	0.81 (0.17)	0.72 (0.21)	0.71 (0.25)
False Alarm Rate				
Negative	0.22 (0.20)	0.23 (0.10)	0.24 (0.21)	0.22 (0.18)
Neutral	0.10 (0.19)	0.06 (0.05)	0.19 (0.33)	0.12 (0.21)
Accuracy Rate				
Negative	0.56 (0.21)	0.55 (0.14)	0.48 (0.29)	0.54 (0.26)
Neutral	0.54 (0.25)	0.59 (0.14)	0.48 (0.28)	0.50 (0.28)
